# Novel Methodologies for Chemical Activation in Organic Synthesis under Solvent-Free Reaction Conditions

**DOI:** 10.3390/molecules25163579

**Published:** 2020-08-06

**Authors:** Claudia Gabriela Avila-Ortiz, Eusebio Juaristi

**Affiliations:** 1 Departamento de Química, Centro de Investigación y de Estudios Avanzados, Av. IPN 2508, 07360 Ciudad de México, Mexico; 2 El Colegio Nacional, Donceles 104, Centro Histórico, 06020 Ciudad de México, Mexico

**Keywords:** solvent-free, mechanochemistry, biocatalysis, microwave activation, photocatalysis, green chemistry, sustainable synthesis

## Abstract

One central challenge for XXI century chemists is the development of sustainable processes that do not represent a risk either to humanity or to the environment. In this regard, the search for more efficient and clean alternatives to achieve the chemical activation of molecules involved in chemical transformations has played a prominent role in recent years. The use of microwave or UV-Vis light irradiation, and mechanochemical activation is already widespread in many laboratories. Nevertheless, an additional condition to achieve “green” processes comes from the point of view of so-called atom economy. The removal of solvents from chemical reactions generally leads to cleaner, more efficient and more economical processes. This review presents several illustrative applications of the use of sustainable protocols in the synthesis of organic compounds under solvent-free reaction conditions.

## 1. Introduction

Classic chemical activation strategies are often associated with highly energy-demanding processes, as well as with the use of solvents that often times are not friendly with the environment and are commonly associated with costly and time-consuming procedures for the preparation and isolation of the desired product.

In recent decades, the search for more environmentally friendly processes constitutes one central theme of research and development in both academic and industrial chemistry. In the 1990s, Anastas and Warner coined the concept of “Green Chemistry” in terms of the design of products and processes that imply the reduction or elimination of substances that are harmful to life or the environment. This philosophy is based on the 12 principles advanced by Anastas and Warner [1].

Following the postulation of the twelve Green Chemistry principles, a significant number of chemists in both academic and industrial settings have focused their efforts on the development of processes that meet those criteria.

Conceptually, one obvious approach to achieve more sustainable processes is by avoiding the use of solvents, since this strategy minimizes the use of chemical auxiliaries while simultaneously decreasing the generation of waste [2]. Indeed, already in 2005, Sheldon argued that “*the best solvent is no solvent*” [3]. Occasionally, this strategy takes advantage of the fact that some of the reactants are actually liquids at ambient temperature, which helps to achieve a homogeneous reaction mixture. Occasionally, a slight excess of one liquid reagent may be used and the process can still be considered solvent-free. In this context, the use of liquid adjuvants or activators acting as reaction medium can be particularly convenient—salient examples are ionic liquids and deep eutectic solvents.

Another aspect that is essential for the eventual fulfillment of the principles of Green Chemistry refers to the chemical activation associated to the reaction of interest. According to conventional methodologies, chemical reactions involving high activation energy require heating for long periods of time that result in high inherent cost and the concomitant risk of decomposition of reagents and/or products. In the last two or three decades, organocatalysis, mechanochemical activation, microwave irradiation, and photocatalysis are among the most effective alternatives for efficient activation under sustainable conditions.

As a topic of great current relevance, Green Chemistry has recently generated a significant number of review articles and monographs describing successful approaches to achieve the activation of chemical transformations in sustainable processes [4,5,6,7,8,9,10,11,12,13,14,15]. The present review focuses on the discussion of several recent applications of non-conventional chemical activation strategies in organic synthesis in the absence of solvents.

## 2. Mechanochemistry

Mechanochemistry is defined as the use of mechanical energy to conduct chemical transformations. The simplest equipment employed to carry out a mechanochemical reaction is the mortar with its pestle, a methodology that can be dated back to the Stone Age. Nevertheless, the first documented mechanochemical reaction is attributed to Theophrastus of Eresus, who was a student of Aristotle. He observed that during the milling process of some cinnabar fragments (HgS) using a copper mortar, drops of a liquid metal (mercury) were produced [16].

**Figure 1:**
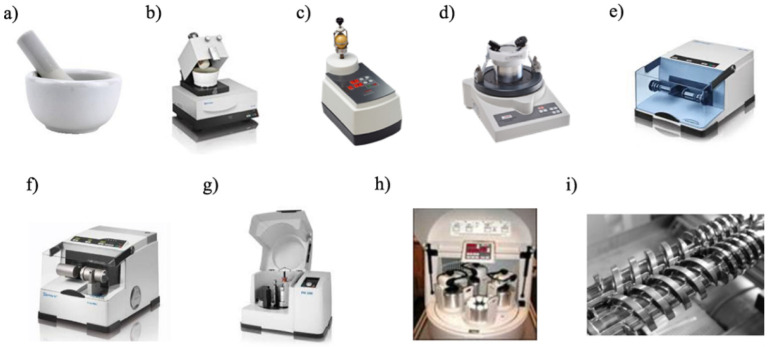
Common grinding and milling apparatus. (**a**) Mortar and pestle. (**b**) Automatic mortar (Retsch). (**c**) Vertical vibrational mini-mill (Fritsch). (**d**) Vibratory micro-mill (Fritsch). (**e**) Vibrational ball-milling (Retsch). (**f**) Vibrational ball-milling with temperature control (Retsch, Cryomill). (**g**) Planetary ball-milling (Retsch). (**h**) Multi-sample milling (Automaxion). (**i**) Twin-screw used for continuous mechanochemical processes. Reproduced with permission of the American Chemical Society from [17].

Currently, there exists various grinding equipment that are already automated for their convenient and reliable use in laboratory (small scale) or industrial (large scale) processes. The most widely used are high speed ball mills (HSBMs), planetary mills and screw mills (Figure 1).

The stress generated by the friction and the knocking of the spheres in the mill reactor give rise to high-temperature microsites in the reaction mixture, promoting the breaking of bonds in the substrate and the formation of new bonds to afford the corresponding products [18].

Among the advantages offered by mechanochemistry, most important is the complete elimination of solvent, or alternatively the use of very small quantities of it as a grinding assistant (i.e., liquid assisted grinding, LAG) [19]. From the above, it is evident that mechanochemistry offers a sustainable alternative for the design of clean and efficient processes in the absence of bulk solvent [20,21,22,23,24,25,26,27,28,29,30].

### 2.1. Catalyzed Reactions under Solvent-Free Ball-Milling Conditions

In the year 2000, the essentially simultaneous publication of the articles by List, Lerner and Barbas [31], on the one hand, and MacMillan and coworkers [32], on the other hand, gave rise to the development of a new area of rather fruitful research in organic chemistry: organocatalysis [33,34,35]. Particularly attractive in this novel field is the demonstration that small organic molecules can catalyze the same chemical reactions that otherwise require large organic biomolecules (enzymes), usually through similar mechanistic pathways. On the other hand, MacMillan’s work conceptualized organocatalysis in an important way by showing its economic, environmental and scientific benefits, while simultaneously describing general activation strategies that could then be applied to a wide range of reactions.

Presently, organocatalysis continues to be a rapidly developing research area, as shown by the large number of publications that have appeared since 2000 [36]. Some recent examples are highlighted in references [37,38,39,40,41,42,43,44,45,46,47,48,49,50,51,52,53,54,55,56]. 

In a particularly interesting development, the combination of mechanochemical activation and organocatalysis has provided a powerful synergistic tool in organic synthesis since it affords unusually efficient processes presenting high yields and remarkable chemo-, regio- and stereoselectivities. Below, some illustrative examples are highlighted.

**Scheme 1:**
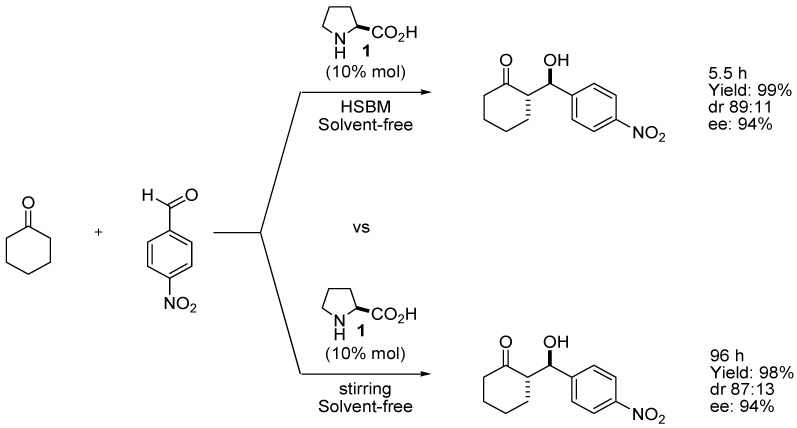
Asymmetric aldol reaction organocatalyzed by (*S*)-proline, **1**. Comparison between conventional stirring in solution and high-speed ball-milling (HSBM) conditions. dr, diastereomeric ratio; ee, enantiomeric excess.

One of the most thoroughly studied organocatalytic reactions is the aldol reaction, since it constitutes one of the most useful strategies for the formation of C-C bonds. In a seminal work reported in 2006, Bolm et al. carried out asymmetric aldol reactions by means of chiral organocatalysts in a ball mill with excellent results [57,58]. Indeed, these experiments demonstrated the advantages of the use of mechanochemistry in enantioselective organocatalyzed aldol reactions, since not only the solvent was removed but the results clearly showed that reaction times were considerably shorter, relative to those in solution (Scheme 1).

**Figure 2:**
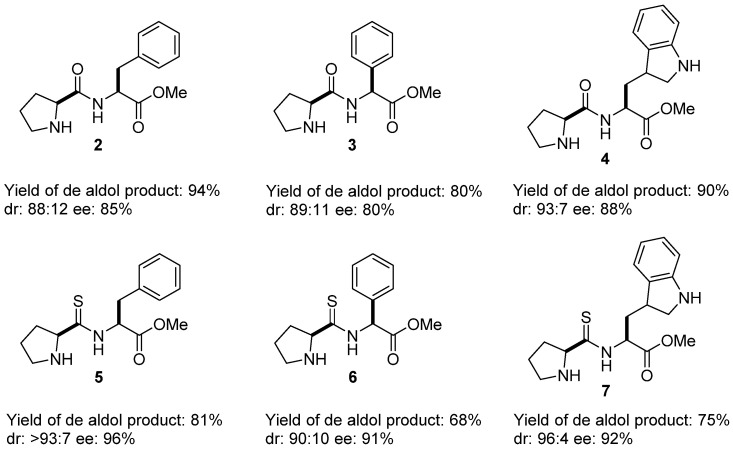
(*S*)-Proline-containing dipeptides **2**–**7** used as organocatalysts for the asymmetric aldol reaction carried out under solvent-free HSBM conditions.

**Figure 3:**
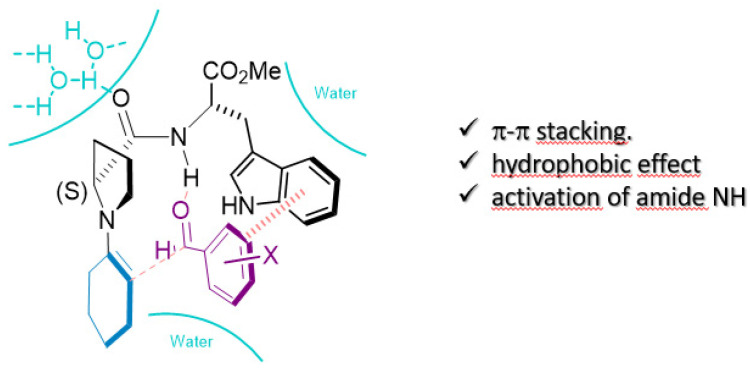
Proposed transition state for the asymmetric aldol reaction between cyclohexanone and *p*-nitro benzaldehyde catalyzed by dipeptide **4**.

Soon thereafter, Hernández and Juaristi found that even higher enantioselectivities were obtained when the reaction depicted in Scheme 1 was catalyzed with dipeptides derived from (*S*)-proline in a HSBM (Figure 2). [59,60]. This observation can be attributed to the fact that dipeptides can be better incorporated into the reaction mixture because they are less polar compounds. Thus, dipeptide **2** showed a catalytic activity superior to that exhibited by proline **1** requiring only 7% catalyst charge [59]. In subsequent studies [60], dipeptides **3** and **4** were compared with **2**, showing that **4** was a better catalyst. Furthermore, the addition of 1.1 equivalents of water increased the catalytic activity of this dipeptide **4** allowing us to reduce the required amount of it down to 3%. These results were explained in terms of a π-π stacking between the aromatic groups present in the catalyst and the aldehyde as illustrated in Figure 3 [60].

The exchange of the carbonyl oxygen atom in dipeptides **2**, **3** and **4** for one sulfur in the thiocarbonyl present in peptides **5**, **6** and **7**, respectively, led to an improvement in the catalytic activity of the thiopeptide analogs. This difference in efficiency is explained in terms of the increased acidity of the N-H hydrogen atom in the thioamide segment, which facilitates the formation of a stronger hydrogen bond and therefore a more robust transition state for the reaction (Figure 3) [61].

As can be observed in Figure 2, the best dipeptidic organocatalysts turn out to be **4** and **5**, whose catalytic activity and efficiency could be improved upon the addition of one equivalent of water to the reaction mixture, together with a catalytic amount of benzoic acid. Apparently, these additives induce the formation of a hydrophobic cavity during the transition state that is further stabilized by π-π stacking between the indole fragment of tryptophan and the aldehyde (Figure 3) [60,62].

Subsequent studies showed, upon comparison with the catalytic efficiency of (*S*)-proline **1** itself, that the chiral site of the second amino acid fragment in dipeptide catalysts does not play a determining role in the stereoselectivity of the reaction. It is, therefore, the proline segment that is responsible for the stereochemistry and selectivity of the aldol product. Nevertheless, the second amino acid residue does improve the catalytic performance of the dipeptide when an aromatic group is present in the side chain as in compounds **1**–**6**; as was previously argued, this is probably a consequence of a π-π interaction with the aromatic ring of the aldehyde [63].

**Scheme 2:**
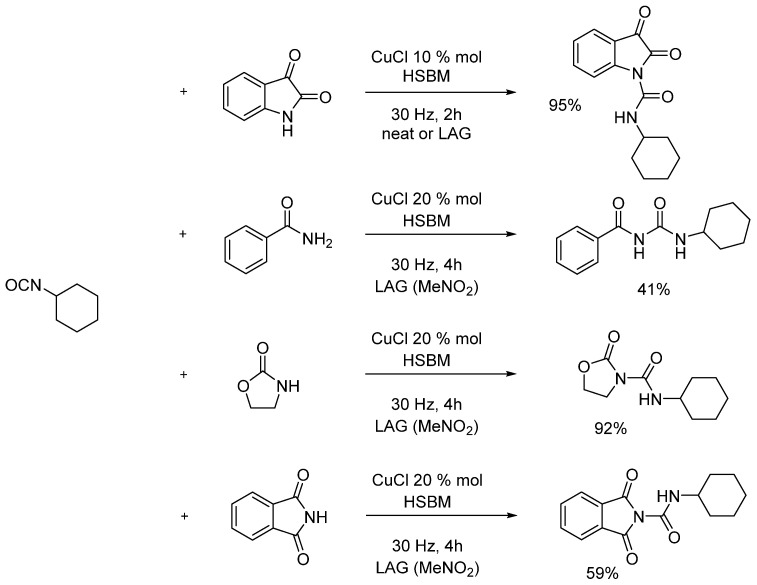
Catalytic nitrogen-carbon coupling reaction using mechanochemistry [64].

Recently, Friščić and collaborators reported the use of ball-milling mechanochemistry to carry out the coupling reaction of isatines, benzamides, imides and phthalimides to isocyanates [64]. This reaction leads to a variety of amide derivatives which are useful in the synthesis of peptides, polymers and pharmacological compounds [65]. In general, when these types of reactions are performed under conventional conditions, they require long reaction times, high temperatures and expensive transition metal catalysts. In Friščić’s work, different copper salts and metallic copper were tested as potential catalysts, revealing that CuCl afforded the best reaction yields. These coupling reactions were carried out in a ball mill at room temperature and under solvent-free conditions (Scheme 2). The use of small quantities of solvent turns out to be crucial for the reaction to take place, since the mixture of the starting materials and CuCl alone does not give any product. The use of nitromethane as a LAG liquid allowed the high-yield preparation of the expected products. This study highlights the power of mechanochemistry as a chemical activation strategy since, when trying the same reactions under conventional conditions, temperatures above 100 °C were required to obtain the desired products in poor yields [64].

In this context, peptide synthesis is presently one of the most salient areas in organic synthesis because of its central relevance in chemistry and biochemistry. Peptides have interesting properties, not just as catalysts for many metabolic reactions but also as pharmacological and biological agents. It is therefore not surprising that a significant number of synthetic chemists are working actively to develop “greener” methodologies for the synthesis of both natural and unnatural peptides [65,66,67,68,69,70,71,72,73]. 

In a pioneering study, Cavani et al. determined the structure and catalytic activity of hydrotalcite Mg-Al minerals with the general formula [Mg^2+^_1−x_Al^3+^_x_(OH)_2_]^x+^(CO_3_^n−^_x/n_)·*m*H_2_O [74]. Among the useful properties of hydrotalcite materials, one can mention their basic character; furthermore, following heat treatment (420–470 °C) they form mixed oxides which are homogeneous. On the other hand, when rehydrated by water vapor, their original layered structure is restored. 

Taking advantage of the properties exhibited by hydrotalcites, in 2016 Landeros and Juaristi reported a convenient strategy for peptide synthesis in the absence of solvent using mechanochemical activation and hydrotalcite as a catalyst [75]. Hydrotalcite (HT-S), its calcined derivative (HT-C), and a reconstructed variant (HT-R) were analyzed as basic catalysts in amino acid coupling reaction to provide a variety of peptides under solvent-free conditions. Preliminary results suggested that the best basic catalyst is Mg-Al hydrotalcite (HT-S) (Table 1, Essay 1).

Once the best reaction conditions had been established, several α- and β-amino acids were employed as substrates and the dipeptides of interest were obtained in good yields as shown in Table 2 [75]. It is important to mention that the catalyst can be recovered and reused with minor loss of its catalytic activity.

**Scheme 3:**
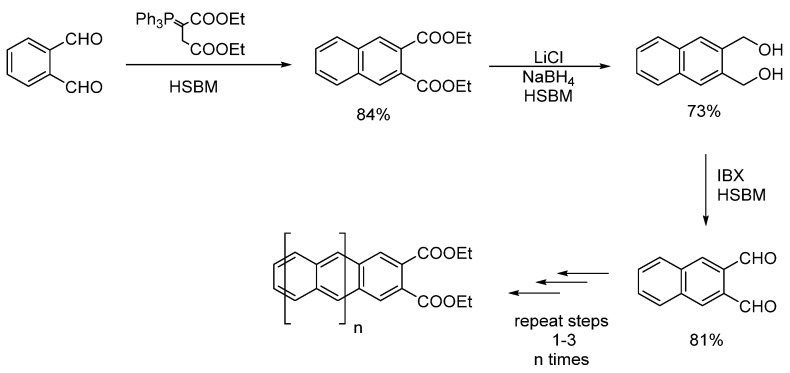
Synthesis of polyaromatic compounds under solvent-free HSBM [76].

In a particularly useful application of mechanochemistry, Mack et al. recently reported a methodology for the synthesis of polyaromatic compounds [76]. These compounds are of interest in synthetic chemistry owing to their application in nanotechnology; nevertheless, their preparation represents a technical and ecological challenge since polyaromatic derivatives are practically insoluble in most solvents. Mechanochemistry offers then the advantage that substrates can be used under solvent-free reaction conditions. Indeed, several of the steps in the synthesis advanced by Mack and coworkers were carried out in a ball mill (HSBM) under solvent-free conditions, making this strategy an environmentally friendly alternative (Scheme 3). 

**Scheme 4:**
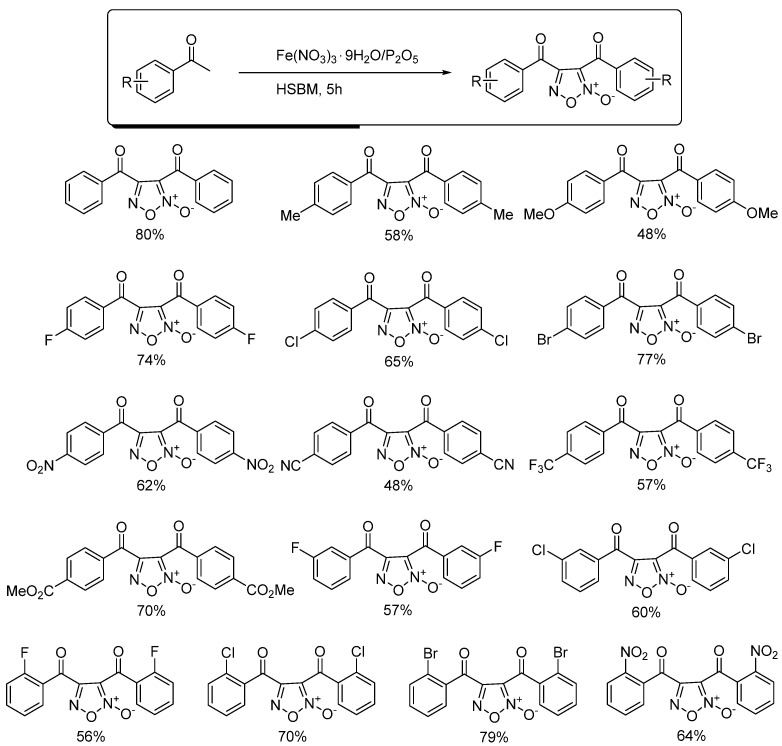
Synthesis of diacylfuroxans under solvent-free HSBM [77].

In this context, compounds of the furaxane type are of interest in organic chemistry because they are synthetic intermediates of molecules with pharmacological activity or with applications in the field of agronomy. In 2019, Guo’s group reported the synthesis of diacyl furoxanes under solvent-free reaction conditions using a high-speed ball mill (Scheme 4) [77]. This methodology uses Fe(NO_3_)_3_∙9H_2_O as a nitrating agent and phosphorous pentoxide as an oxidant. The authors tested different nitrate salts but Fe(NO_3_)_3_∙9H_2_O afforded the highest yields. The scope of these reactions includes acetophenones which are substituted with either donor or electron withdrawing groups, and the expected products were obtained in moderate to good yields as shown in Scheme 4.

A multicomponent reaction is one where three or more reagents combine to afford a new product. This type of reaction is important from the sustainability point of view due to their high atom economy, as well as the elimination of several reaction steps that would require isolation and purification protocols. Multicomponent reactions reflect also the state of the art in chemical control since generally C-C and C-X (X = heteroatom) bonds are formed in one single step. For recent interesting applications of mechanochemistry in multicomponent reactions see references [78,79,80,81,82,83,84,85,86,87].

**Scheme 5:**
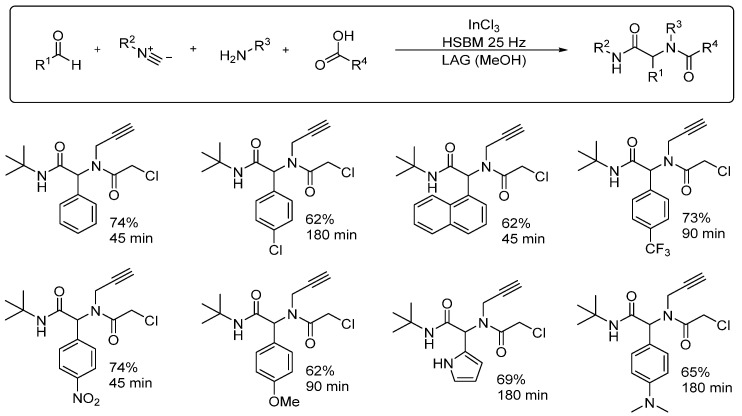
Ugi 4-CR Reaction under solvent-free and HSBM conditions [88].

In 2016, Polindara-García and Juaristi reported a methodology for the Ugi 4-CR and the Passerini 3-CR multicomponent reactions in the absence of solvent using a ball mill. [88]. In the case of the Ugi 4-CR, the energy provided by the ball mill was sufficient to afford the products of interest. When comparing the results with those of reactions carried out in solution under normal heating or even microwave conditions, it is concluded that mechanochemistry is a better synthetic strategy since it shortened the reaction times and afforded better yields for a variety of substrates as shown in Scheme 5.

**Scheme 6:**
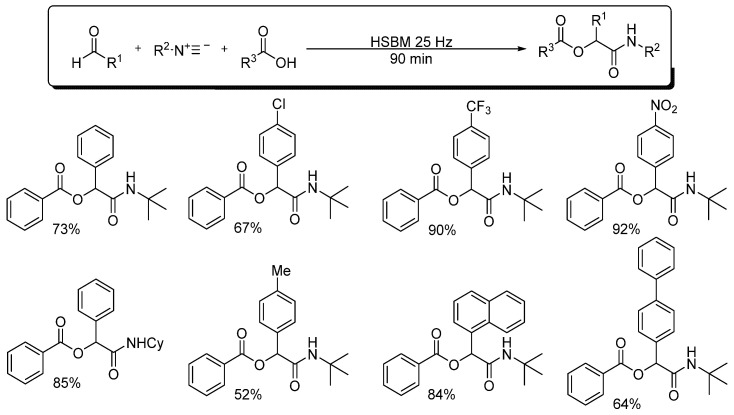
Passerini 3-CR reaction under solvent-free HSBM conditions [88].

Equally good results were obtained with the Passerini 3-CR reaction. It is important to note that in the Passerini 3-CR reaction, the heat generated within the mill reactor was sufficient to promote the reaction without the need for additional energy input, and that the reaction times are shorter than those required under conventional reflux conditions in solution, affording the desired products in moderate to excellent yields (Scheme 6).

**Scheme 7:**
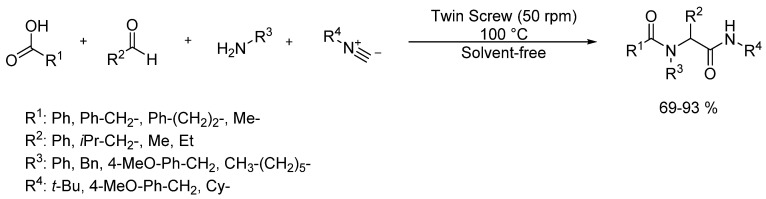
Ugi reaction under solvent-free conditions using a twin-screw reactor [89].

Very recently, Eldahy’s group reported a methodology for the Ugi 4-CR reaction in a twin-screw grinding system without catalyst and without solvent [89]. The reaction proceeded with good yields at a temperature of 100 °C (Scheme 7).

**Scheme 8:**
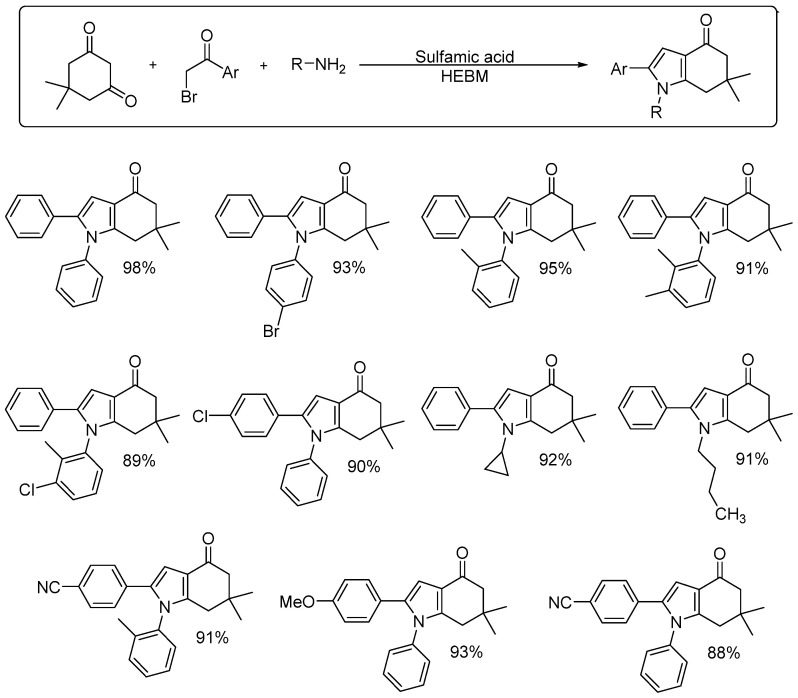
Synthesis of 4-oxo-tetrahydroindoles with solvent-free HEBM [90].

In 2019, Lambat and Banerjee developed a methodology for the synthesis of 4-oxo-tetrahydroindoles using a solvent-free, multicomponent one-pot reaction in a high energy ball mill (HEBM) [90]. Sulphamic acid was used as the catalyst; it was easily isolated and recovered from the reaction mixture and reused up to five times with the same efficiency (Scheme 8).

### 2.2. Enzymatic Reactions under Solvent-Free Ball-Milling Conditions

Enzymes are conveniently used as catalysts because they present a large number of advantages (see below), they operate at room temperature, are highly selective, and promote a wide variety of reactions [91,92,93,94,95]. Processes involving enzymes are considered green and sustainable because they comply with at least 10 of the 12 principles of Green Chemistry; that is:

- Waste generation is minimized;
- Atom economy is maximized;
- Formation of toxic products is avoided;
- Functional and safe products are produced;
- The use of auxiliary substances is minimized;
- Energy consumption is reduced;
- Renewable materials are employed;
- The use of derivatives is eliminated or reduced;
- Catalytic reagents are employed;
- Biodegradable products are usually obtained.

Indeed, enzymes are naturally available molecules representing non-toxic reagents, and biodegradable molecules [96]. Due to their high efficiency and versatility, enzymes (in particular lipases) have been used both in small-scale settings (e.g., laboratories) and large-scale synthetic industrial pharmaceutical processes for the preparation of a wide variety of organic compounds. The necessary synthetic steps that may be assigned to enzymes can involve hydrolytic, enantioselective, condensation reactions, and even multicomponent reactions [97,98,99,100]. These processes can be carried out on scales of several grams in either batch or continuous flow reactors [101].

Although it may seem counterintuitive, enzymes present significant resistance in unconventional media such as ionic liquids [102], deep eutectic solvents [103] and ball-milling apparatus (cf. mechanoenzymatic methodologies) [104]. Below, we will present some examples of the combined use of enzymes and mechanochemistry in organic synthesis.

**Scheme 9:**
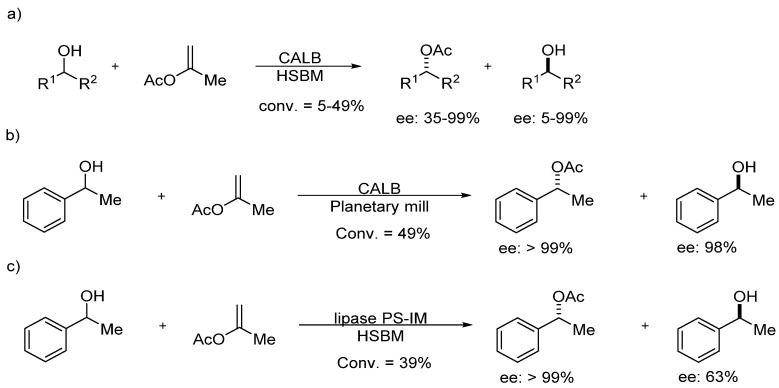
Mechanoenzymatic resolution of secondary alcohols with CALB. (**a**) Separation of racemic 1-phenylethanol. (**b**) Process carried out on a gram scale. (**c**) Process carried out with the immobilized enzyme lipase PS-IM [105].

The first report disclosing the use of enzymes in mechanochemistry was the one revealed by Hernández et al. who described the mechanoenzymatic resolution of secondary alcohols [105]. When carrying out the enantioselective resolution of racemic carbinols with *Candida antarctica* Lipase B (CALB) enzyme in a ball mill (HSBM), Hernández and coworkers obtained excellent results. For instance, for the separation of 1-phenylethanol a conversion = 47% (out of a theoretical maximum of 50%), with enantiomeric excess (ee) higher than 99% for the acetate product, and ee = 90% for the recovered alcohol (Scheme 9a). The wide scope of the method was verified by the good results obtained for a family of racemic secondary alcohols that could be resolved through this methodology. Not only that, the process could be carried out on a gram scale in a planetary mill, obtaining practically the same conversion and selectivity (Scheme 9b). An additional bonus is that the enzyme could be recovered and reused up to three more cycles with some loss in the degree of conversion but maintaining an excellent enantioselectivity for the acetylated product. By contrast, when the resolution process was carried out with the immobilized enzyme lipase PS-IM, the products of interest were obtained with less efficiency in terms of conversion and enantiomeric excess in the recovered alcohol (Scheme 9c).

**Scheme 10:**
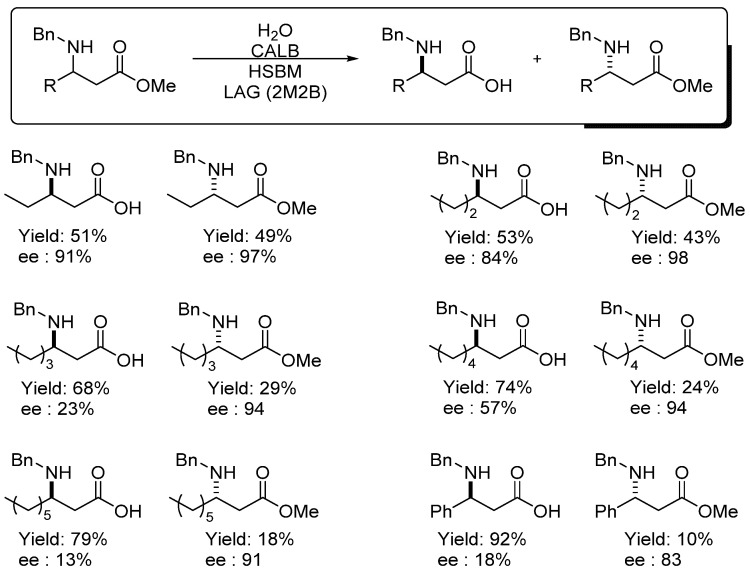
Enzymatic resolution of *N*-benzylated-β^3^-amino esters under solvent-free HSBM conditions [106].

Soon thereafter, Pérez-Venegas et al. reported a methodology for the enzymatic resolution of *N*-benzylated-β^3^-amino esters using *Candida antarctica* lipase B in a ball mill (HSBM) [106,107]. The resolution process took place essentially in the absence of solvent with only a half equivalent of water added to the reaction mixture and 0.2 mL of 2-methyl-2-butanol (2M2B) as the liquid grinding assistant (LAG). The reaction proceeded at ambient temperature; that is, no additional heating was required. The yields achieved in this mechanoenzymatic approach were comparable to those obtained in solution [108] but conveniently with a significant reduction in reaction times. Furthermore, the process could be scaled up one order of magnitude and the enzyme catalyst could be recovered and reused twice, maintaining the enantiomeric excess of the products, although with a decrease in yield due to partial denaturation of the enzyme and partial destruction of the support (Scheme 10).

**Scheme 11:**
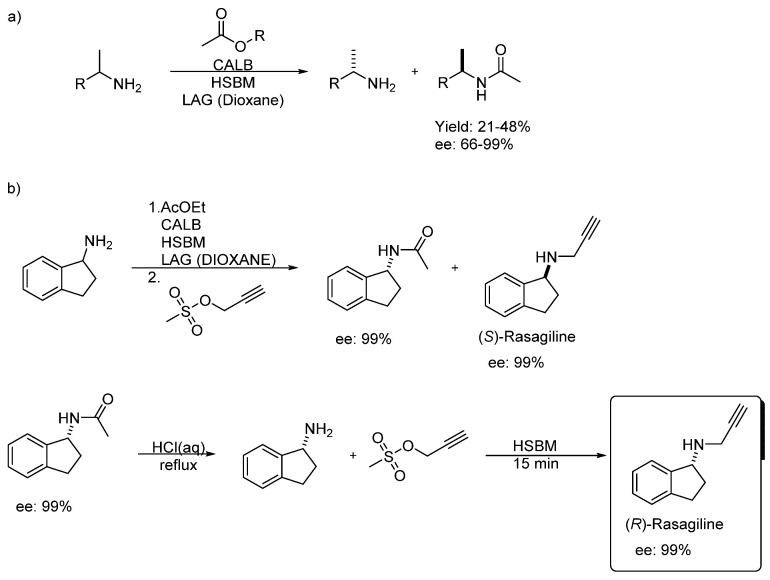
(**a**) Mechanoenzymatic resolution of chiral amines and (**b**) synthesis of both enantiomers of Rasagiline [109].

Subsequently, Pérez-Venegas and Juaristi reported the mechanoenzymatic resolution of chiral amines [109], including the enantioselective synthesis of (*R*)-Rasagiline (a drug employed for the treatment of Parkinson’s disease) and its (*S*) enantiomer. In this case, the resolution process required dioxane as a grinding adjuvant (Scheme 11a). Following the initial enantioselective acylation reaction of the (*S*) enantiomer, the addition of propargyl mesylate to the reaction mixture afforded (*S*)-Rasagiline (Scheme 11b). On the other hand, (*R*)-Rasagiline was prepared by acid hydrolysis of the acylated product followed by treatment with propargyl mesylate in the ball mill (Scheme 11b). Chiral amines are important building blocks for the synthesis of molecules with pharmacological activity. According to this mechanoenzymatic procedure, it was possible to obtain a family of chiral amines with high enantiomeric purity under solvent-free conditions.

As was mentioned above, peptide synthesis continues to be one of the areas of greatest interest in organic chemistry. This is due to the fact that peptides have many diverse applications, not only in the synthesis of organic compounds, but most importantly in the food and pharmaceutical industry [110,111,112]. In this regard, in recent years mechanochemistry has played an important role in the green synthesis of these compounds [103,104,105,106,107,108,109,110,111,112,113,114,115,116,117,118].

**Scheme 12:**
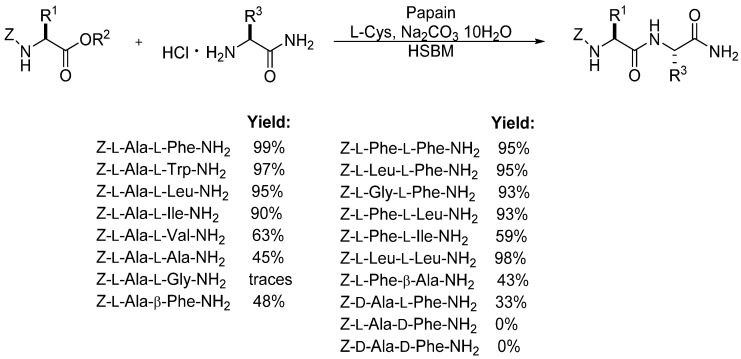
Mechanoenzymatic synthesis of dipeptides with Papain [119].

In 2017, Bolm’s group reported the mechanoenzymatic synthesis of dipeptides using the enzyme Papain in a HSBM reactor [119]. The results obtained from this research proved the versatility of the mechanoenzymatic method since it allowed the high yield preparation of a variety of dipeptides, including those containing amino acids with steric impediment, which are generally difficult to couple, such as valine and isoleucine. It was also possible to prepare α,β-dipeptides containing the β-alanine residue (Scheme 12). By contrast, it was observed that the enzyme is not capable of incorporating *d*-amino acids. The reproducibility of the process was verified in a planetary mill and in a twin-screw reactor [120]. 

**Scheme 13:**
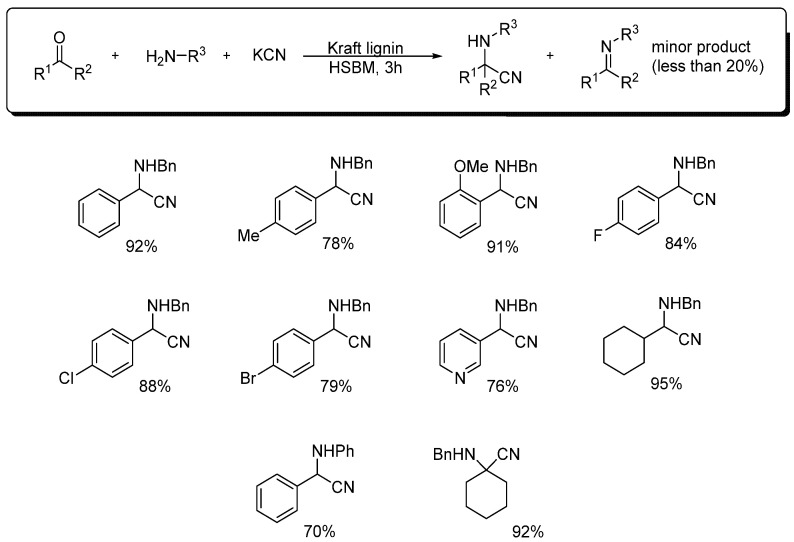
Strecker reaction promoted by Kraft lignin under solvent-free HSBM conditions [121].

Furthermore, in 2017, Hernández et al. reported the Strecker reaction between aldehydes and ketones with amines catalyzed with lignin under solvent-free conditions and HSBM to obtain α-aminonitriles [121]. The reaction proceeds well, and the yields are good to excellent with the minimum presence of the imine obtained as a by-product of the reaction (Scheme 13). The study compared peanut shell powder, cellulose and lignin as promoters of the Strecker reaction, the latter showing the best catalytic activity.

**Scheme 14:**
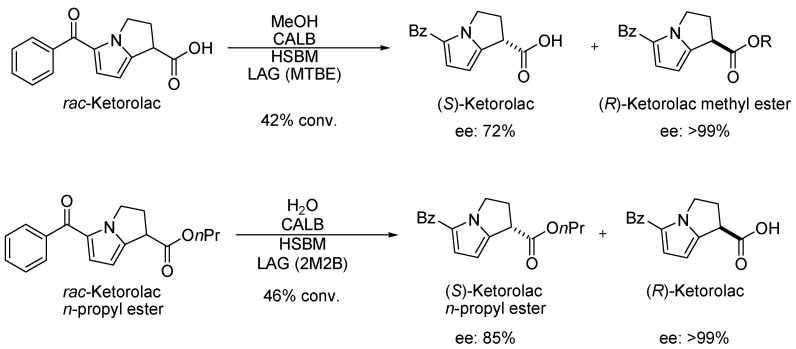
Dual mechanoenzymatic resolution of Ketorolac under solvent-free HSBM conditions [122].

Very recently, Juaristi and coworkers reported a dual mechanoenzymatic resolution of Ketorolac with CALB enzyme [122]. This strategy allows for the isolation of both enantiomers of the drug with high enantiomeric purity, either through enantiomeric esterification of the racemic Ketorolac carboxylic acid with methanol, or via the enantioselective hydrolysis of its racemic *n*-propyl ester (Scheme 14). Both Ketorolac enantiomers are valuable because of their pharmacological activity. (*S*)-Ketorolac is a powerful pain reliever, while its enantiomer (*R*)-Ketorolac is used in the treatment of ovarian cancer.

## 3. Microwave Activation under Solvent-Free Conditions

Microwave irradiation has been revolutionizing organic chemistry for over a couple of decades. Just as in home cooking, instant heating and optimization of reaction times are the main attractive feature of this form of activation [123]. Among the advantages offered by the use of microwaves in organic synthesis, are better yields and higher purity of the reaction products, energy saving, uniform heating and good reproducibility [123,124]. Due to the above, the use of microwave activation is now quite widespread among groups working in the area of organic synthesis, generating a large number of publications [125,126,127,128,129,130,131].

One of the potential problems in the employment of microwave reactors is the occasional explosion of the reactor flasks or tubes owing to the pressure exerted by the reaction solvent during irradiation. For this reason, the elimination of solvent has become rather important, which actually leads to shorter reaction times and better yields [124]. Below there are some illustrative examples of the use of microwaves in solvent-free reactions.

**Scheme 15:**
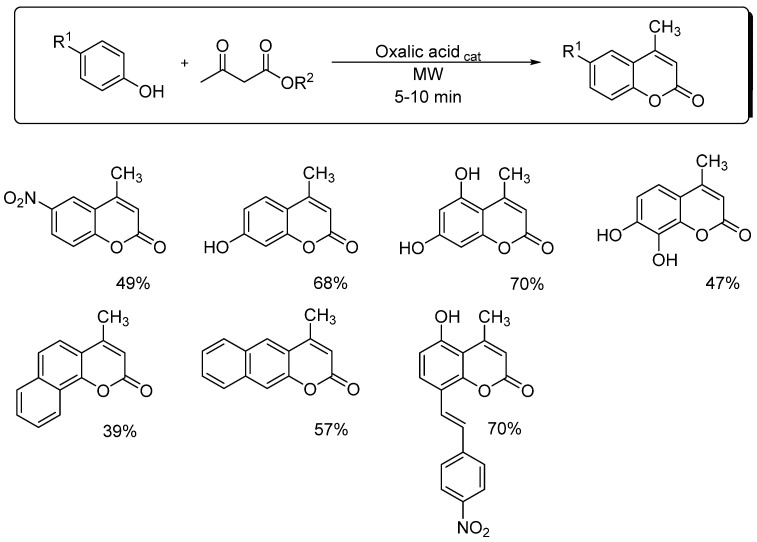
Solvent-free synthesis of coumarins under microwave irradiation [132].

In 2017, Monga et al. reported the synthesis of coumarins using microwaves in a solvent-free process [132]. Coumarins are compounds found in a variety of plants and have interesting therapeutic properties. This procedure uses oxalic acid in catalytic amounts and allows the preparation of a variety of products that demonstrate the scope of the method (Scheme 15).

**Scheme 16:**
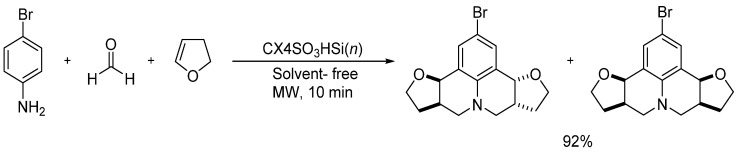
Microwave assisted solvent-free synthesis of Julolidines [133].

In this context, Fernandes’ group published in 2019 a convenient synthesis of Julolidines by means of a microwave-assisted supported catalysis in the absence of solvent [133]. This strategy involves a multicomponent reaction as shown in Scheme 16. The supported catalyst is a Calix[4]arene that showed synergistic catalytic activity when acting together with microwave activation in a metal-free process. In addition, the easy removal of the catalyst simplifies the isolation of the products (both diastereomers of the Julolidines of interest) that were obtained in an approximate 1:1 ratio. Julolidines are a family of compounds of relevance owing to their application as antivirals, antidepressants, as well as for their useful properties in non-linear optics [134,135].

**Scheme 17:**
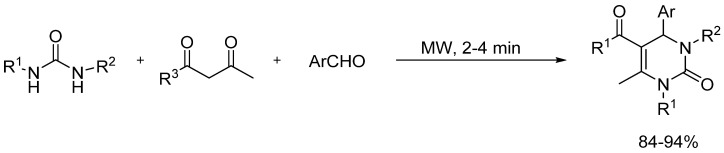
Solvent-free Biginelli reaction using microwave irradiation [136].

An additional application of microwave activation under solvent-free conditions was described in 2019 by Rao et al., who reported the multicomponent microwave-assisted preparation of Biginelli derivatives in the absence of solvent [136]. This reaction proceeds in just a few minutes with yields in the range of 84–94% as shown in Scheme 17.

**Scheme 18:**
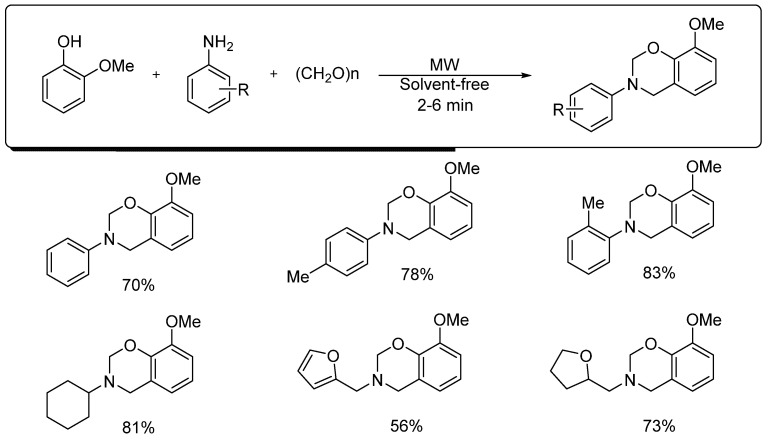
Microwave synthesis of benzoxazines under solvent-free conditions [137].

In another interesting example, in 2017 Lomonaco et al. reported the synthesis of benzoxazines by means of an environment-friendly microwave-assisted methodology in the absence of solvent [137]. Benzoxazines are useful intermediates in the manufacture of resins employed in the area ofpolymers and synthetic materials. This work presents the synthesis of a family of compounds obtained with short reaction times of 2–6 min with moderate to good yields as shown in Scheme 18.

## 4. Photocatalysis under Solvent-Free Conditions

The use of light as an energy source for chemical activation has boomed in recent years. It is certainly a rapidly growing research area that has largely been promoted by the availability of efficient light sources, which are cheap, with precise wavelengths, and quite powerful (e.g., light emitting diodes, LED) [138]. Photocatalysis with visible light has multiple applications in the synthesis of organic compounds via cross coupling reactions, cycloadditions, fluorination, etc. When comparing these procedures with conventional ones, one finds that photocatalysis frequently promotes reactions under milder reaction conditions, such as at ambient temperature [139]. Photocatalysis generally depends on the use of catalytic amounts of metallic complexes (that are toxic and expensive) or on organic pigments that may not be recyclable [140]. As a consequence, researchers in synthetic organic chemistry work day by day in the development of processes that are friendly to the environment and that may be achieved through photo-organocatalytic processes; that is, by the use of small molecules that interact with light promoting chemical reactions [141]. Photocatalysis is therefore a topic of current interest and has given rise to many publications [142,143,144]. However, there are very few reports of the use of this technique under solvent-free conditions.

**Scheme 19:**
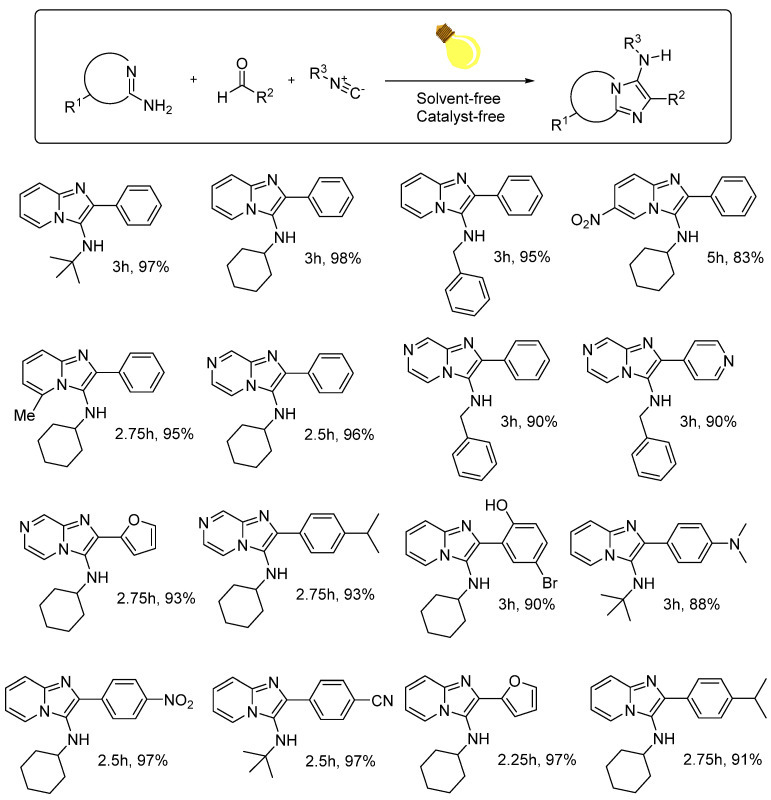
Photocatalytic synthesis of imidazol pyridines and imidazol thiazoles under solvent-free conditions. The yellow bulb-shaped sign indicates irradiation with visible light [145].

In the year 2018, Siddiqui and collaborators described a photocatalyzed methodology for the synthesis of imidazole pyridines and imidazole thiazoles under solvent-free conditions and without the need for a catalyst, using only visible light as an activator (Scheme 19) [145].

Imidazol pyridines and imidazol thiazoles are of interest as intermediaries in drug synthesis. The methodology reported by the Siddiqui’s group is a simple and “green” alternative because it does not require catalysts or solvent. It is important to remember that photochemical reactions often require metal complexes as catalysts. The reported yields for a variety of substrates range from 83 to 98%, which demonstrates the usefulness of the method.

**Scheme 20:**
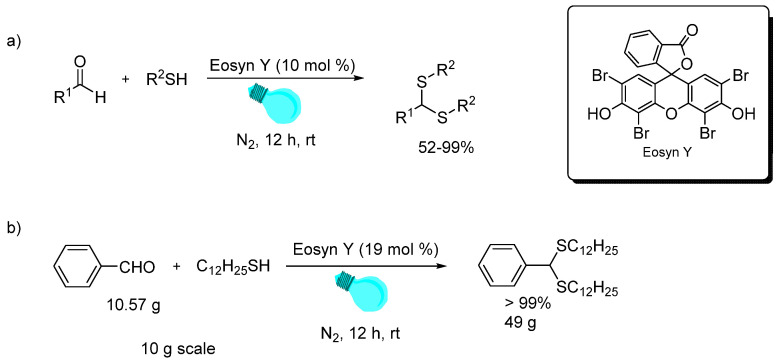
(**a**) Photocatalytic thioacetalization of aldehydes under solvent-free conditions. (**b**) Scaling-up of the process to obtain 49 g of the reaction product. The blue bulb-shaped sign indicates irradiation with blue LED light [146].

In this context, Lee’s group recently reported the photochemical solvent-free reaction of aldehydes with thiols in the synthesis of thioacetals. The reaction is catalyzed by Eosyn Y and activated by blue LED light at ambient temperature with good yields (Scheme 20) [146]. This work was highlighted by scaling of the process to obtain 49 g of the reaction product between benzaldehyde and dodecanothiol (Scheme 20b).

**Scheme 21:**
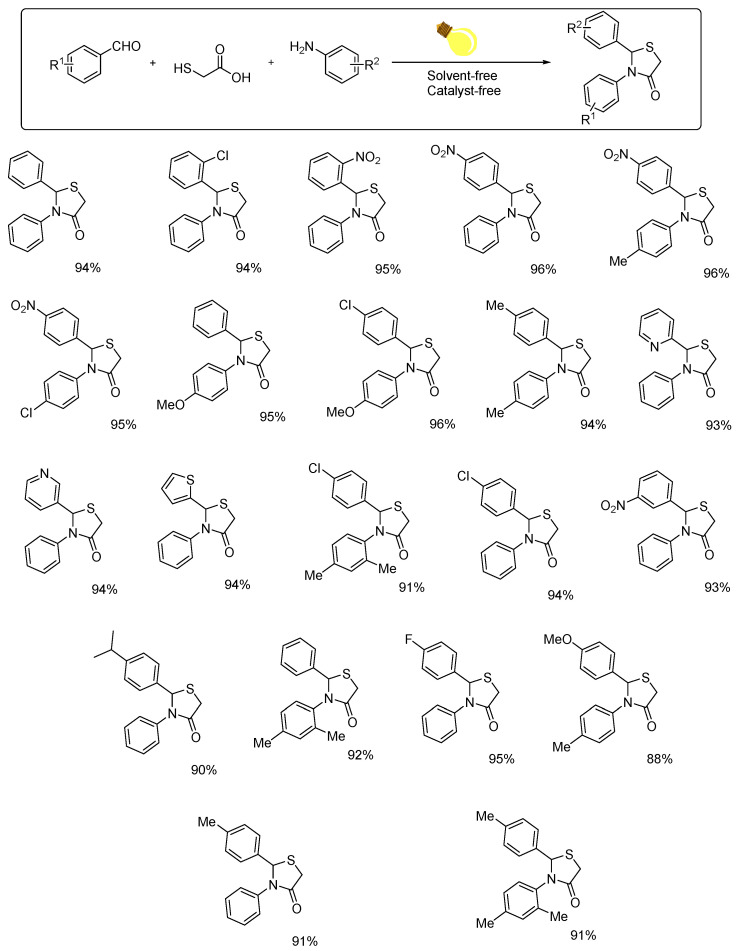
Photocatalytic synthesis of 1,3-Thiazolidin-4-ones under solvent-free conditions. The yellow bulb-shaped sign indicates irradiation with visible light [147].

Very recently, Siddiqui reported the photocatalytic synthesis of 1,3-thiazolidin-4-ones in the absence of a solvent [147]. This is another multicomponent reaction that proceeds under photochemical activation without the need of a metal catalyst. The scope of this technique afforded excellent results (all yields exceeding 90%) for a variety of substrates (Scheme 21). The protocol is superior, relative to those previously reported that required the use of toxic and expensive metal catalysts, high temperatures, and the use of solvent.

In conclusion, the search for processes that comply with the principles of “Green Chemistry” has motivated researchers in organic synthesis to develop efficient alternatives to carry out the chemical activation of numerous synthetic processes. Among the most profitable alternatives are solvent-free mechanochemistry, microwave activation, photocatalysis and the combination of enzymatic catalysis with mechanochemical activation. The reactions carried out according to these methodologies usually afford good yields, take place under mild reaction conditions and offer the possibility of scale-up processes. It is true that from a strict point of view, the isolation of products and the subsequent purification procedures often require column chromatography or at least filtration followed by recrystallization, involving necessarily the use of solvent. Nevertheless, non-traditional activation strategies such as mechanochemistry, microwave activation and photochemistry, when carried out in the absence of solvent, certainly result in more efficient synthetic procedures and contribute significantly to the overall atom economy, waste minimization and sustainability of the process.

## Figures and Tables

**Table 1:**
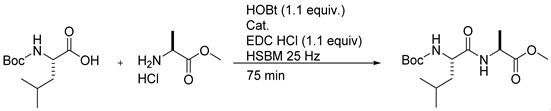
Effect of the catalyst (base) in the coupling reaction of amino acids activated by HSBM [75].

Essay	Catalyst (Base)	[mg]	Yield [%]
1	HT-S	250	89
2	HT-C	250	55
3	HT-R	250	89
4	NaHCO_3_	250	76
5	Cs_2_CO_3_	250	67
6	K_2_CO_3_	250	15

**Table 2:**
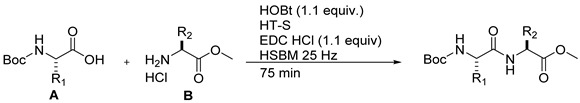
Scope of the coupling reaction of amino acids catalyzed by Mg-Al hydrotalcite [75].

Essay	Amino Acid A	Amino Acid B	Dipeptide Product	Yield (%)
1	Boc-Leu	Ala	Boc-Leu-Ala	89
2	Boc-β-ala	Phe	Boc-β-Ala-Phe	89
3	Boc-β-ala	Val	Boc-β-Ala-Val	85
4	Boc-β-ala	Ala	Boc-β-Ala-Ala	79
5	Boc-β-ala	Leu	Boc-β-Ala-Leu	89
6	Boc-β-ala	Ile	Boc-β-Ala-Ile	82
7	Boc-β-ala	Gly	Boc-β-Ala-Gly	78
8	Boc-β-ala	His	Boc-β-Ala-His	70
9	Boc-β-ala	β-Ala	Boc-β-Ala-β-Ala	73
10	Cbz-Phe	Phe	Cbz-Phe-Phe	83
11	Cbz-Phe	Ala	Cbz-Phe-Ala	85
12	Fmoc-Val	Phe	Fmoc-Val-Phe	83
13	Fmoc-Val	Ala	Fmoc-Val-Ala	88
